# Digital footprints and recruitment: an experimental study on the impact of social media content on hiring decisions

**DOI:** 10.3389/fpsyg.2025.1693850

**Published:** 2025-10-28

**Authors:** Nazlı Türker, Engin Üngüren

**Affiliations:** Department of Business Management, Faculty of Economics, Administrative and Social Sciences, Alanya Alaaddin Keykubat University, Antalya, Türkiye

**Keywords:** social media, professional competence, person–organization fit, hiring intention, signaling theory, experimental research

## Abstract

**Introduction:**

The aim of this study is to reveal the extent to which and the ways in which the content that candidates share on their social media profiles influences the evaluations of decision-makers in recruitment processes.

**Methods:**

To that end, an experimental study was conducted with 480 managers and human resources specialists authorized in recruitment processes at four- and five-star hotels in different regions of Türkiye. A fictional scenario was developed to create a realistic recruitment situation. Participants were randomly assigned to a control group and two experimental groups, where they examined résumés of candidates together with social media profiles, manipulated to appear either professional or non-professional, and evaluated their hiring intentions. Thus, the study tested the weight of personal signals perceived from social media posts, alongside the professional competencies and experience stated in the candidates’ résumés, in the decision-making processes of evaluators.

**Results:**

The findings show that social media content significantly influences perceptions of professional competence and person–organization fit, thereby altering hiring intention. In particular, negative social media content was found to overshadow the professional competence signal, even for highly qualified candidates, leading to a prioritization of perceived cultural fit. In contrast, professional content enabled candidates to send a positive fit signal, supporting hiring intention.

**Discussion and conclusion:**

Social media profiles function as a strategic moderator that reinforces or weakens the technical signals presented in résumés and substantially shapes the perceived suitability of candidates. These results indicate that social media content is not only a supplementary source of information but also a strong signaling mechanism that guides recruitment decisions. The study makes theoretical contributions to Signaling Theory and provides important practical implications regarding the use of social media in recruitment processes.

## 1 Introduction

Recruitment processes have long been an important research topic in the academic literature as one of the fundamental and critical functions of strategic human resource management in organizations (Barney and Wright, 1998; Ahmad and Schroeder, 2002; Tipper, 2004; DeCenzo et al., 2017; Melão and Reis, 2020). The view that human resources are the fundamental factor influencing the success of organizations shows that the recruitment process involves not only identifying candidates with the right competencies but also selecting individuals who align with the organization’s culture and goals (Koch and McGrath, 1996; Barney and Wright, 1998; Pfeffer and Veiga, 1999; Ahmad and Schroeder, 2002; Ployhart and Weekley, 2010; Tang et al., 2021). Especially since employees play a decisive role in achieving the strategic goals of the organization, the recruitment process has been extensively examined in both theoretical and practical terms in organizational behavior and human resource management literature (Markoulli et al., 2017; Nikolaou, 2021). In this context, the effectiveness and validity of candidate selection methods, as well as their impact on both candidates and organizations, remain current and debated issues today.

With the rise of digitalization in the business world, social media platforms have moved beyond being mere channels for individuals to engage in social interaction and share information, becoming important sources from which employers obtain professional information about candidates (Brown and Vaughn, 2011; Berkelaar, 2014). Many existing studies show that organizations widely use social media platforms (e.g., Facebook, LinkedIn, X/Twitter) to make more holistic evaluations of candidates (Kluemper and Rosen, 2009; Clark and Roberts, 2010; Caers and Castelyns, 2011; Berkelaar, 2014; Zide et al., 2014; Kluemper et al., 2016; Baert, 2018; Koch et al., 2018; Rosen et al., 2018; Hosain and Liu, 2019). Thus, social media has increasingly become a tool for not only assessing candidates’ technical competencies but also for evaluating their social, cultural, and personality characteristics (Sameen and Cornelius, 2013; Nikolaou, 2014; Bohmova and Pavlicek, 2015; Alexander et al., 2019).

The integration of social media into recruitment processes is referred to in the literature as “cybervetting” and has been gaining importance as an increasingly systematic practice (Berkelaar and Buzzanell, 2014). Also called online screening, this process involves employers collecting personal and professional information about candidates from social media and other online sources and taking this information into account in recruitment decisions (Berkelaar, 2017; McDonald et al., 2022; Wilcox et al., 2022; Akbulut et al., 2024; Hoover et al., 2025). For example, a study conducted by the Society for Human Resource Management (2016) reported that approximately 43% of employers in the United States examine social media profiles during recruitment processes and that 36% have eliminated candidates based on such reviews (McDonald et al., 2022).

Posts, photographs, events attended, social relationships, interests, and personal information not included in résumés that appear on social media profiles are carefully examined by employers, and this information can influence a candidate’s likelihood of being hired either positively or negatively (Williams and Almand, 2014; Roth et al., 2016). According to Jobvite (2014) Social Recruiting Survey, 93% of employers stated that they review social media profiles to support a candidate’s employability. Similarly, Society for Human Resource Management (2016) and CareerBuilder (2017) reports indicate that social media posts can lead to candidates being eliminated during the evaluation process, and that a large proportion of employers are influenced by the information they obtain about candidates through social media. These findings show that social media has begun to play an important role in recruitment processes and is regarded as a complementary element alongside traditional method (Lawhern, 2016).

Traditionally, first impressions were formed during face-to-face interviews based on a candidate’s attire, manner of speaking, and behavior; however, in today’s digital world, candidates’ online profiles have also become one of the main determinants of these impressions. Information obtained through social media can provide important clues not only about a candidate’s professional identity but also about their social, cultural, and ethical values. However, this also raised new debates concerning ethics, privacy, bias, and discrimination. The significance of this study lies in its examination of the impact of social media on recruitment decisions through an experimental method. The study aims to reveal empirically how individuals authorized to make recruitment decisions evaluate candidates’ résumés and social media profiles together, and how information obtained from social media affects recruitment decisions. In this context, the central research questions of this study are as follows:

- How does information obtained from social media influence evaluators’ perceptions of candidates’ professional competence and person–organization fit?
- Does the nature of social media content (positive/negative) play a moderating role in the relationship between résumé-based signals and hiring intention?

Although this research is based on findings obtained in the Turkish context, the impact of social media on recruitment processes can be regarded as a dynamic with global relevance. The transformation brought about by digitalization in working life affects hiring decisions in similar ways across different cultures and industries. For instance, Society for Human Resource Management (2016) reports in the United States, Hedenus et al. (2021) in Europe, and Kwok and Muñiz (2021) in Asia have demonstrated that social media has increasingly become a tool used in candidate evaluations.

The theoretical foundation of the study is based on Spence’s (1973) Signaling Theory. According to this theory, employers infer unobservable attributes (e.g., ethical values, person–organization fit) from candidates’ observable characteristics (e.g., social media profiles) (Bangerter et al., 2012; Piopiunik et al., 2020; Oostrom et al., 2021). The existing literature has shown that social media provides employers with strong signals about candidates’ personality traits (Kluemper and Rosen, 2009), abilities (Berkelaar, 2014), and organizational fit (Roulin and Bangerter, 2013). For example, Kluemper and Rosen (2009) found that information derived from Facebook profiles influenced personality assessments, while Roulin and Bangerter (2013) suggested that social media serves as a source of signals for assessing candidates’ professional qualities and person–job fit. Similarly, Van Iddekinge et al. (2016) demonstrated that recruiters’ use of social media information led to significant differences in evaluations across demographic groups (e.g., female and white applicants).

This study seeks to extend the current literature in two important ways. First, it empirically tests the moderating role of social media content, examining how it strengthens or weakens the relationship between candidates’ signals of professional competence and person–organization fit and their perceived hiring intentions. Second, it contributes to the literature by investigating how evaluators process multiple signal sources—résumés and social media—and which source they prioritize when signals are conflicting. Therefore, this study not only confirms that social media functions as a signal source but also examines how these signals interact with traditional signals in shaping recruitment decisions.

## 2 Literature review and hypothesis development

### 2.1 Factors influencing hiring decision

The systematic evaluation of candidates during the recruitment process is critically important for both meeting the qualifications required for the position and ensuring long-term organizational fit. The success of this evaluation stage directly affects labor productivity and overall organizational performance through the selection of the right candidate (Kristof-Brown et al., 2005). The literature identifies numerous criteria that shape hiring intention; among these, professional competence, person–organization fit, communication skills, leadership potential, teamwork orientation, problem-solving ability, and general social skills stand out (Kristof-Brown et al., 2005; Ployhart, 2006). However, the variables of professional competence and person–organization fit have a decisive impact on decision-makers (Roulin and Bangerter, 2013; Stanišić and Čerović, 2020). Through an experimental design, this study examines the relative weight of these two factors on hiring intention and how social media content reframes this relationship.

#### 2.1.1 The effect of professional competence on hiring intentions

One of the fundamental topics debated in the management literature is the question of what constitutes the most critical factor determining job performance. The origins of this debate date back to Antiquity. From Plato’s inquiries into competence and job performance in the 4th century BC (Mulder et al., 2007; Primoff and Sidney, 1988) to Taylor’s (1911) methods for increasing worker productivity through the scientific management approach developed after the Industrial Revolution, a substantial body of literature has emerged. Taylor, in particular, emphasized the central role of individuals’ competencies in achieving effective job performance and demonstrated, through time and motion studies, how these competencies could be measured and managed (Sandberg and Pinnington, 2009).

Professional competence is not limited to technical knowledge and skills; it encompasses a wide range of them, from general job abilities to social competencies. Recent studies also show that the qualifications sought by employers in candidates have diversified and increased. For example, in a comprehensive content analysis study conducted in the Slovak tourism sector, Derco and Tometzová (2023) found that employers value not only technical skills but also social skills (e.g., communication, teamwork) and language competencies. Particularly, these social skills, often referred to as “soft skills,” have been shown to serve as important signals that differentiate candidates from others.

Similarly, Marneros et al. (2021), in their study conducted in the hospitality sector, modeled professional competence through six main dimensions: leadership and critical thinking, information technology and financial analysis, human relations and communication, interpersonal communication and cultural diversity, human resources management, professional image, and operational knowledge. The results of the study showed that not only technical competencies, but also managerial, cultural, and interpersonal skills have a strong impact on the likelihood of being hired. In particular, people management skills and practical professional knowledge stand out among the decisive criteria in employers’ hiring decisions. In another study conducted in Latvia by Līce and Sloka (2019), findings from 750 companies revealed that employers place greater emphasis during the recruitment process on candidates’ attitudes, self-management, and social competencies, while professional knowledge and experience are considered following these elements. These findings indicate that in the modern business world, it is no longer enough for candidates to be technically competent alone; instead, a more holistic competency profile is required.

In the selection of senior executives, professional competence is regarded as an indispensable factor. In the decision support software developed for the case of hotels and travel agencies, Stanišić and Čerović (2020) found that the most important selection criterion was professional competence, followed by the ability to plan work, teamwork, and foreign language skills. This result suggests that as job complexity increases, the priority of professional competence also rises. Another noteworthy dimension on this issue is indicated in Behrenz’s (2001) study conducted in Sweden. Within the framework of Signaling Theory, the study revealed that employers initially focus on candidates’ education level and experience during the application process, while in the interview stage they place importance on professional knowledge, commitment, and social competencies. This finding confirms that the professional competence signal is important in both the pre-screening and detailed evaluation stages of the recruitment process.

The education and work experience stated in the résumé provides employers with signals about a candidate’s potential performance (Spence, 1973). Employers seek to predict candidates’ future success by evaluating their professional competencies. Especially under conditions of uncertainty, the most concrete and measurable signal a candidate can offer is their professional knowledge and skills. For employers, education and professional competencies stand out as primary factors assessed before less observable characteristics such as personality traits and values (Connelly et al., 2011). However, it is also observed that employers look not only at technical competencies but also at how effectively these competencies can be applied in the organizational context. This requires assessing the extent to which professional competencies align with organizational culture and job requirements. Therefore, employers focus not only on whether candidates possess knowledge but also on their capacity to effectively use that knowledge. Especially in the service and tourism sectors, alongside technical knowledge, the ability to manage human relations comes to the forefront (Marneros et al., 2021; Derco and Tometzová, 2023). Findings in the literature show that professional competencies have a clear effect on hiring intention. Competencies such as candidates’ education level, work experience, and technical and social skills are among the elements most considered by employers when assessing their suitability for a position. When competence signals are clear, concrete, and reliable, they contribute to the development of a favorable inclination toward the candidate in the employer’s decision-making process. In this context, we propose the following hypothesis.

*H1*: Candidates’ professional competence affects evaluators’ hiring intentions.

#### 2.1.2 The effect of person-organization fit on hiring intentions

Person–organization fit refers to the degree of alignment between employees’ individual characteristics and the values, culture, and goals of the organization, and it has been a concept of interest in the management literature for many years (Kristof, 1996; Sekiguchi, 2004). Research shows that low person–organization fit leads to decreases in employee motivation, job satisfaction, and organizational commitment; increases in stress and organizational cynicism; and consequently, a higher tendency to leave the job (Ivancevich and Matteson, 1980; Matteson and Ivancevich, 1982; O'Reilly et al., 1991; Vancouver and Schmitt, 1991; Cable and Judge, 1994; Edwards, 1996; Greguras and Diefendorff, 2009; Leung and Chaturvedi, 2011; Peng and Mao, 2015; Teimouri et al., 2015; Jin et al., 2018; Memon et al., 2018; Hamstra et al., 2019; Ko and Campbell, 2021; Kakar et al., 2022). In contrast, the literature emphasizes that a high level of person–organization fit provides positive effects at both individual and organizational levels (Sekiguchi, 2004; Greguras and Diefendorff, 2009).

In today’s professional world, not only candidates with high competencies, experience, and knowledge are preferred; but also, those who embrace the organization’s vision, mission, and values. This is because it is not sufficient for an employee to merely fulfill the requirements of the job; they are also expected to adapt to the cultural composition of the organization, build positive social relationships in the workplace, and identify with organizational goals. For this reason, human resources departments pay attention in recruitment processes to the candidates’ personal values and behaviors and whether or not they align with the distinctive characteristics of the organization in addition to their technical qualifications (Aktaş, 2011). While professional competence in recruitment processes can be easily assessed through tangible indicators such as degrees and certificates, compatibility with organizational culture is perceived largely through abstract elements such as personality, attitudes, and values, making it relatively more difficult to evaluate, yet critically important (Koçak, 2023). Therefore, employers seek to understand person–organization fit by being attentive of not only candidates’ technical knowledge and skills but also to various characteristics such as hobbies, personal preferences, social relationships, communication style, and even appearance. This approach goes beyond the person–job fit focus of traditional human resources practices and offers a more holistic perspective on how a person will contribute to the organizational context (Cheng, 2014).

In a study conducted in the tourism sector, Barakazı (2023) showed that human resources managers consider not only candidates’ job qualifications but also their alignment with the company’s goals and values. The study found that candidates who sent signals of compatibility with the company’s vision and culture were evaluated more positively. Similarly, in a study conducted in the hospitality sector by Kwok et al. (2011), person–organization fit and person–job fit emerged among the most important factors that recruitment specialists considered when evaluating new graduates. For employers, according to the study, it is not only the matching of the job description that matters but also sharing the organization’s values and contributing to its social environment, which are among the key criteria influencing candidate selection.

In recent years, social media has become an important source for evaluating signals about candidates’ person–organization fit. In this context, studies by Roulin and Bangerter (2013) and Zide (2015) show that social media profiles reflect not only candidates’ professional competencies but also their values, social behaviors, and potential for cultural fit. Social media reveals a candidate’s interests, social attitudes, communication style, and professional image, giving employers the opportunity to assess whether the person is suitable not only for the job requirements but also for the organization’s social and cultural structure. For example, candidates with professional and well-maintained social media profiles are generally perceived by employers as having high fit potential, whereas those who post inappropriate content or display attitudes contrary to corporate values are evaluated more negatively.

All these findings imply that person–organization fit is a strong determinant of evaluators’ hiring intentions. With the addition of new information sources such as social media, information about candidates’ personality traits and values has become more visible and easier to evaluate. In light of these explanations, we propose the following hypothesis.

*H2*: Candidates’ person-organization fit affect evaluators’ hiring intentions.

### 2.2 The effect of social media content on hiring decisions

Social media has emerged as a central tool in recruitment processes. In addition to traditional résumés and references, social media profiles offer recruitment specialists the opportunity to gain a more comprehensive understanding of candidates (Cuizon, 2019; Van Dijck, 2013). In particular, candidates’ behavior on social media platforms, the content they share, and the comments they make provide important clues about their personalities, values, and compatibility with organizational culture. In this context, Quirdi’s (2016, as cited in Denizli, 2020, p. 105) statement, “While your paper résumé tells what you have done, your social résumé reflects who you are,” underlines why social media has become so influential in hiring decisions. Similarly, Roberts and Roach (2009) note that social media profiles offer far more personal information than résumés, while Hedenus et al. (2021) state that this information is related to the candidate’s professional life. In addition, Saylin and Horrocks (2013, as cited in Nurata, 2023) show that social media provides information that cannot be obtained through application forms and interviews.

The impact of social media content on recruitment decisions is also supported by empirical studies. For example, in a CareerBuilder (2017) survey, approximately 48% of recruitment specialists reported finding elements in candidates’ social media profiles that could lead to their elimination. The most common negative elements included inappropriate or provocative photographs (46%), content related to alcohol and drug use (43%), discriminatory comments (33%), negative statements about previous employers (31%), and poor communication skills (29%). On the other hand, factors on social media that supported positive hiring decisions were listed as a professional image, compatibility with the organization’s culture, diverse interests, positive personality traits, and effective communication skills (Özkum, 2018). Van Iddekinge et al. (2016) also found that social media profiles containing disrespectful and inappropriate content negatively affected candidates’ evaluation scores.

Various experimental studies also highlight the role of social media content in recruitment processes. Williams and Almand (2014) found that candidates with strong résumés, but social media profiles filled with inappropriate content had difficulty securing interview opportunities. Similarly, Henderson (2019) noted that posts related to alcohol, drugs, and sexual content were perceived by employers as “red flags.” Özkum (2018) found that posts on social media involving breaches of confidentiality about previous employers and discriminatory content reduced the likelihood of being hired, while culturally appropriate and job-related content had a positive effect. Existing studies also show that the impact of social media content is not limited to negative signals. Alarcon et al. (2019) stated that positive information on social media increased the likelihood of being preferred regardless of the candidate’s qualifications. Baert (2018) showed that even a social media profile photo could influence hiring decisions, with candidates having more attractive profiles being invited to interviews more often. Bohnert and Ross (2010) also found that family-oriented or professional content was evaluated more positively than alcohol-related content.

Other studies supporting these findings have also examined which characteristics stand out in hiring decisions on social media platforms. For example, Kwok and Muñiz (2021) found that managers in the hospitality sector predominantly looked for signals of extroversion, leadership, and professionalism on LinkedIn profiles, while on Facebook, avoiding inappropriate language and content was emphasized. Nugroho and Trinugroho (2018) classified social media content as professional and non-professional and showed that both types were decisive in hiring decisions.

Focusing on the tourism sector, Demir and Günaydın (2023) stated that human resources managers see social media as an effective reference source for gathering information about candidates. Hedenus et al. (2021) showed that employers use social media to assess a candidate’s reliability and social capital, while McDonald et al. (2022) argued that social media reviews are important for reducing risks and increasing organizational fit.

Social media profiles provide evaluators with rich information not only about a candidate’s technical competencies but also about their personality traits, values, and compatibility with organizational culture. In particular, platforms such as Facebook and Instagram are used to assess personal and social fit, while LinkedIn is used to evaluate professional competence (Sameen and Cornelius, 2013; Hoek et al., 2016). In this context, social media enables candidates to convey to employers not only their past performance but also their potential behaviors and values.

Findings in the literature show that social media content can send both positive and negative signals, and that these signals directly influence hiring decisions (Kluemper and Rosen, 2009; Özkum, 2018; Becton et al., 2019). Information on candidates’ social media profiles such as posts, group memberships, social circles, liked pages, and expressed political views, provides important clues about the candidate and shapes evaluators’ decisions (Chang and Madera, 2012; Madera, 2012). As a result, social media content enables employers to consider not only a candidate’s technical competencies but also their personality, values, and fit with organizational culture in the recruitment process. The professional and personal information offered by social media platforms creates differentiation among candidates during evaluation processes and significantly affects their chances of being hired. In this context, the following hypothesis is proposed:

*H3*: Evaluators’ assessments of candidates’ professional competence and person–organization fit differ depending on the candidates’ social media content.

People send and receive numerous signals every day; these signals are conveyed to others through speech, behavior, appearance, and interactions (Karasek and Bryant, 2012). Interpreting these signals is important in decision-making processes. Signaling theory is a key theoretical framework developed to explain how people use observable indicators (signals) when making decisions under uncertainty. In the labor market, employers evaluate various signals from candidates when deciding whether to hire them, while candidates, either consciously or unconsciously, produce signals to demonstrate their competencies and fit (Connelly et al., 2011).

Signaling theory forms the main theoretical foundation of this study. In Job Market Signaling, Spence (1973) emphasized that employers have incomplete information about candidates’ competencies and that hiring is an investment decision. Spence argued that during recruitment, evaluators must distinguish between information that can be directly observed about a candidate (indices, e.g., gender, age) and information that cannot be observed but is conveyed by the candidate (signals, e.g., education, experience). According to Spence, candidates present alterable signals such as education and experience to introduce themselves to employers, while employers interpret these signals to minimize their risks. Thus, signaling theory helps reduce the problem of asymmetric information (Connelly et al., 2011, p. 40).

An important extension of signaling theory in the recruitment context is that the signals presented by candidates are not limited to résumés and references but are also conveyed through social media profiles. The recruitment literature increasingly recognizes the role of social media platforms, and signaling theory provides a powerful framework for understanding this phenomenon. Roulin and Bangerter (2013) have shown that social media platforms have become a new source of signals in this regard. Similarly, Kluemper and Rosen (2009) demonstrated that information obtained from social media profiles can provide valid signals about personality traits, while Van Iddekinge et al. (2016) highlighted the potential risks of negative effects stemming from such signals. In this context, social media can generate signals that either complement or contradict résumé-based qualifications, thereby directly shaping employers’ evaluation processes.

An experimental study conducted by Piopiunik et al. (2020), found that cognitive and social skill signals in different résumés had significant effects on the likelihood of being invited to an interview. In particular, information technology and foreign language skills in female candidates were perceived by employers as important signals. Similarly, Oostrom et al. (2021) showed that inappropriate clothing style sent a low-status signal, reducing the likelihood of being hired, although strong professional qualifications could partly compensate for this negative signal. The study demonstrated that the impression evaluators form is shaped by a combination of the qualifications stated in résumés and the content shared on social media.

These results show that social media content functions not only as supplementary information but also as a signal that guides the evaluation process. Employers draw inferences from social media profile content about both the candidate’s level of professional competence and their fit with organizational culture. Thus, social media stands out as a platform capable of producing both positive and negative signals. Given that people are cognitively inclined to make quick decisions (Karasek and Bryant, 2012), employers may generalize a single positive or negative element on a candidate’s social media profile to their entire decision. In this context, social media content functions as a strong source of signals but may also jeopardize the accuracy of decisions. Therefore, candidates’ social media content plays a critical role in shaping evaluators’ perceptions and can strengthen or weaken the effects of professional competence and person–organization fit signals.

Signaling theory offers an important framework for understanding how content shared on social media is evaluated and how this content influences hiring intention. In particular, Roulin and Bangerter (2013) have shown that social media content shapes employers’ perceptions of candidates and produces meaningful signals that can affect hiring decisions. Therefore, it can be said that social media content not only provides information about a candidate’s competencies but also reveals the alignment of their values and behaviors with organizational culture. Based on the findings in the literature, the fourth hypothesis of the study is formulated as follows:

*H4*: The effect of candidates’ professional competence and person–organization fit on hiring intention changes significantly depending on the nature of the candidates’ social media posts (social media content plays a moderating role).

## 3 Methods

### 3.1 Sample and procedure

A true experimental method was employed in this study to rigorously test the causal relationships between variables. Experimental methods, particularly due to their high internal validity designs, have the capacity to produce the strongest findings in terms of cause–effect relationships (Fraenkel et al., 2012). The research was conducted using the Post-Test Only Control Group Design. The main feature of this design is the random assignment of subjects to experimental and control groups, allowing for the comparison of the effects of the treatment applied to the experimental group on the dependent variable. In this context, participants were randomly assigned to Control, Experimental-1, and Experimental-2 groups. The treatment was applied only to the experimental groups, while the control group was left under natural conditions. True experimental designs ensure high internal validity through random assignment of groups and control of external variables; thus, any differences obtained can be causally attributed to the treatment (Rubin and Babbie, 2011, as cited in Gürbüz and Şahin, 2018).

Participants were assigned the role of a recruiter responsible for the hiring process of a “Sales and Marketing Manager” position in a hotel located in Alanya, which had been operating for five years. The scenario content was developed using job descriptions and qualifications derived from real advertisements published on major career portals in Türkiye, thereby enhancing ecological validity. In order to further strengthen the scenario’s representativeness of real hiring intentions, after the scenario participants were asked to respond on a 5-point Likert scale to the items: “This scenario reflects a real hiring situation” and “My decision felt like making an actual recruitment decision.” Evaluation forms with a mean score below 4 were excluded from the study. To measure the research variables, widely used and psychometrically validated Likert-type scales from the literature were employed. In the control group, only the résumés of the two candidates were evaluated, whereas in the experimental groups, in addition to the résumés, Facebook-based social media profiles of the candidates (professional vs. non-professional) were presented. These profiles were constructed by incorporating elements known in the literature to generate positive or negative hiring signals (e.g., inappropriate language, alcohol/party-related content, negative job-related statements versus professional achievements, volunteerism, and work-related posts). In compliance with the Turkish Law on the Protection of Personal Data (KVKK), no real profiles were used; instead, standardized stimulus sets were created using MetaHuman characters and royalty-free images. To minimize demographic bias, candidates were designed with comparable age and gender characteristics.

The sample of the study consisted of business owners, general managers, assistant general managers, human resources managers, and recruitment specialists authorized to hire in four- and five-star accommodation enterprises operating across all regions of Türkiye, particularly in the Mediterranean, Aegean, and Marmara regions. The study was conducted between March 2023 and September 2023. Taking into account the recommendations in experimental and factorial designs that 20–30 subjects per experimental condition are sufficient (Gravetter and Forzano, 2013; as cited in Başgöze and İşkorkutan, 2020), a total of 480 participants were obtained. Participants were assigned to the Control, Experimental 1, and Experimental 2 groups through computer-assisted randomization. The experimental design of the study is presented in Table 1.

**Table 1:** The experimental design.

Groups	Procedure	Variables	Post-test
R-C	X_a_ + X_b_	Y_1_, Y_2_, Y_3_	O_1_
R-E_1_	X_a_ + X_b_ + X_a1_ + X_b1_	Y_1_, Y_2_, Y_3_	O_2_
R-E_2_	X_a_ + X_b_ + X_a2_ + X_b2_	Y_1_, Y_2_, Y_3_	O_3_

R-C = Random Control Group, R-E_1_ = Random Experimental Group 1, R-E_2_ = Random Experimental Group 2, X_a_ = Candidate A Résumé, X_b_ = Candidate B Résumé, X_a1_ = Non-Professional Social Media Content, X_b1_ = Professional Social Media Content; X_a2_ = Professional Social Media Content, X_b2_ = Non-Professional Social Media Content, Y_1_ = Professional Competence, Y_2_ = Person-Organization Fit, Y_3_ = Hiring Intention, O_1_ = Random Control Group Post-Test, O_2_ = Random Experimental Group 1 Post-Test, O_3_ = Random Experimental Group 2 Post-Test.

Before the start of the study, a small-scale pilot test was conducted on the questionnaire form for the Experimental-2 condition, reaching 50 specialists. The pilot data confirmed that all item formulations were clear and that the scales were applicable; therefore, no revisions to the measurement instruments were needed before proceeding with the main study. In terms of research ethics, ethical approval was obtained from Alanya Alaaddin Keykubat University’s Social and Human Sciences Scientific Research Ethics Committee on 07.12.2022, with decision number 202217, prior to data collection. The 50 participants in the pilot study were not included in the main study and were used solely to test the validity of the scales. During the sampling process, difficulties were encountered in reaching participants, particularly outside the Mediterranean Region, so data collection in these areas was carried out through a research consultancy firm. Between March and May 2023, the firm delivered the questionnaires to four- and five-star accommodation enterprises, reaching a total of 280 authorized managers. In contrast, in the Mediterranean Region, data collection was conducted directly by the researchers between June and September 2023 through face-to-face surveys during on-site visits to the enterprises, obtaining data from 200 participants. To test whether the two data collection channels created any difference in response quality, an independent samples t-test was conducted, showing no statistically significant difference between the two groups (*p* > 0.05). This multi-stage approach ensured that the targeted sample size was achieved and increased representativeness across different geographical regions.

### 3.2 Research procedure

#### 3.2.1 Control group

Participants in the control group were presented with the résumés of two applicants. The first, referred to as “Candidate A,” was designed as an applicant with standard qualifications. The second, referred to as “Candidate B,” was designed as an applicant with relatively high-level competencies. The neutral naming of the candidates was intended to prevent potential perceptual or cultural associations and biases. In addition, personal information such as address and phone number were removed from the résumés, which were arranged to allow examination solely of professional qualifications.

The résumés of Candidate A and Candidate B were systematically differentiated in terms of education level, work experience, foreign language proficiency, completed courses and certifications, computer skills, association and club memberships, and hobbies. Candidate A, with standard qualifications, had worked exclusively in the Alanya region for many years, spoke only one foreign language (English), had no international experience, and presented an average level of foreign language and computer skills. In contrast, Candidate B, with high qualifications, had graduated from schools providing foreign language education since high school, completed a master’s degree abroad in sales and marketing management, gained international work experience, spoke advanced-level German and French in addition to English, had completed numerous professional courses and certifications, possessed strong computer and programming skills, engaged in remarkable hobbies such as scuba diving and paragliding, and maintained active memberships in associations and clubs related to the field of work.

Participants in the control group were presented only with the candidates’ résumés. They were then asked to evaluate the candidates and provide their opinions on each candidate’s professional competence, person–organization fit, and hiring intention. In this process, no social media information or any other external data was provided; evaluations were based solely on the résumés. Thus, the data obtained from the control group reflected assessments made exclusively on the basis of official résumé information. This approach allowed for the identification of decision-making processes based solely on traditional information sets, providing a point of comparison with the experimental groups, where the effects of additional information sources such as social media content were tested.

#### 3.2.2 Experimental groups

In the study, two separate experimental groups were formed: Experimental 1 and Experimental 2. As in the control group, participants in the experimental groups were presented with the candidates’ résumés; however, in addition, they were also shown two different fictional social media profiles created for each candidate. Participants were provided with instructions containing the necessary information about the process and were asked to make their evaluations based on both the résumés and the social media profiles. The social media profiles of the candidates were prepared on the Facebook platform. Facebook was chosen because it remains the most widely used social media platform both globally and in Türkiye (We Are Social, 2023). Moreover, previous studies in the literature have also indicated that Facebook and LinkedIn are the social media platforms most frequently used in recruitment processes (Roulin and Bangerter, 2013; Melanthiou et al., 2015; Hoek et al., 2016).

The candidates’ social media profiles were structured by taking into account photos, personal information and posts, comments from friends, number of followers, and the information provided in their résumés. Of the two profiles prepared, one was designed to contain more professional and positive content. The professional and positive profile reflected the candidate’s professional achievements, participation in social responsibility projects, involvement in sports activities, and positive attitudes toward work life. Posts included content related to job success, awards, supportive comments from their social circle, and a professional demeanor. In contrast, the other social media profile was prepared to include more negative signals. In this profile, the candidate appeared to focus heavily on nightlife, occasionally used inappropriate language, and had friends’ comments reflecting the same tone. In addition, the candidate openly shared dissatisfaction about work life on social media, presenting an unprofessional image. The number of followers was set considerably higher than that of the professional profile.

In preparing the social media profiles, various studies in the literature were used as a reference. Especially those presenting findings that elements such as alcohol consumption, inappropriate language, and negative posts about work have adverse effects on evaluators’ decisions guided this process (Bohnert and Ross, 2010; Hartwell, 2015; Wade, 2015; Özkum, 2018; Becton et al., 2019; Zhang et al., 2020). In contrast to what is frequently seen in the existing studies, this study did not use real individuals’ social media profiles. The reason for this was to comply with ethical and legal obligations under the Personal Data Protection Law in Türkiye. Therefore, in creating the social media profiles, support was obtained from a social media specialist; MetaHuman characters were developed in line with the researcher’s suggestions, and photographs of these characters were used in the profiles. Other photographs were obtained from non-copyrighted sources and adapted to be consistent with the MetaHuman characters. All information and posts included in the profiles were fictionalized and checked by the researcher.

In addition, since the literature indicates that gender can influence evaluators’ hiring decisions (Becton et al., 2019), care was taken to match the two candidates in terms of age and gender. Both candidates were designed as male characters between the ages of 35 and 40, with no information provided regarding marital status. This ensured that variables such as gender, age, and marital status were controlled, allowing the effect of social media content to be observed more clearly. In this way, groups Experimental 1 and Experimental 2 evaluated both the résumés and the social media profiles of the candidates and expressed their opinions on the candidates’ professional competence, person–organization fit, and hiring intention. This procedure allowed for a comparison of the potential impact of social media information on hiring decisions with the control group.

In the Experimental 1 group, Candidate A, who had standard qualifications, was assigned a social media profile that conveyed an unprofessional, negative impression. This profile depicted the candidate as leisure-oriented, having low work engagement, using inappropriate language, and posting negative comments about work life. On the other hand, Candidate B, who had high qualifications, was assigned a professional social media profile. In the Experimental 2 group, participants were also presented with the résumés and social media profiles of the two candidates. In this group, Candidate A, with standard qualifications, was given a professional social media profile, whereas Candidate B, with high qualifications, was assigned a social media profile that conveyed an unprofessional, negative impression. In both experimental groups, participants completed a questionnaire and provided their evaluations regarding the candidates’ professional competence, person–organization fit, and hiring intention after reviewing their résumés and social media profiles.

### 3.3 Measurement

Scales with proven validity and reliability in the literature were used to measure the dependent and independent variables. To assess participants’ evaluations of candidates’ professional competence, the *Professional Competence Evaluation Scale* developed by Üngüren and Türker (2023) was used. The scale consists of items related to sector experience, managerial experience, foreign language proficiency, computer skills, certifications, education level, and fields of education. Items were scored on a five-point Likert-type scale (1 = Not Competent at All, 5 = Highly Competent). To determine participants’ perceptions of how well candidates fit with the organization, the four-item *Perceived Person–Organization Fit Scale* (*α* = 0.96) developed by Kristof-Brown (2000) based on Cable and Judge’s (1997) work was used. Items were evaluated on a five-point Likert-type scale (1 = Not Suitable at All, 5 = Completely Suitable). To measure participants’ hiring intentions toward the candidates, a three-item scale from Cable and Judge’s (1997) study was adapted. The scale consists of three dimensions: the recommendation decision to hire the candidate, the likelihood of success in the job, and the overall evaluation. Items were rated on a five-point Likert-type scale (1 = Strongly Disagree, 5 = Strongly Agree). Higher scores indicate a higher hiring intention for the candidate. Additionally, a form developed by the researcher was used to gather participants’ socio-demographic information and social media habits. The form included questions on sex, age, education level, managerial experience, length of employment in the organization, the organization’s field of activity, position, and region, as well as the presence of a social media account, membership status, frequency of social media use, and the use of social media tools in the recruitment process.

### 3.4 Data analysis

For the statistical analysis of the data obtained in the study, SPSS 26 and SmartPLS 4 software packages were utilized. The analysis process was carried out in two stages. In the first stage, the reliability and validity of the measurement model were tested. In this context, construct reliability was evaluated through Cronbach’s Alpha (α) and Composite Reliability (CR) values, followed by the assessment of convergent validity and discriminant validity. In the second stage, the analysis of the structural model was performed. Path coefficients (*β*) and their statistical significance levels for the hypothesized relationships in the research model were tested using the Bootstrapping method (5,000 resamples). In addition, the explanatory power of the model was examined by analyzing the coefficient of determination (*R*^2^), effect size (*f*^2^), and predictive relevance (*Q*^2^) values. To compare the control and experimental groups, independent sample t-tests and one-way analysis of variance (ANOVA) were applied. Finally, in order to examine the moderating role of social media content, the PLS-MGA (Partial Least Squares – Multi-Group Analysis) approach was employed.

## 4 Findings

### 4.1 Demographic findings

A total of 480 people participated in the study, and participants were randomly assigned to three groups. According to the findings in Table 2, the Control Group consisted of 177 participants, Experimental-1 Group consisted of 147 participants, and Experimental-2 Group consisted of 156 participants. The distribution of participants across groups appears relatively homogeneous in terms of group size. In terms of sex, in all three groups, female participants constituted more than half; however, the proportion of male participants was close to that of females. Regarding age distribution, the majority of participants were in the 28–37 age range. When level of education was examined, in all three groups most participants had bachelor’s degrees. In terms of length of employment in the organization, the highest proportion belonged to those who had been employed for 1–4 years, followed by those employed for 5–8 years. Examining the distribution by job position, in all three groups the most common positions were human resources manager and human resources specialist. The distribution of participants in other positions was also similar across groups. Geographically, participants working in the Mediterranean Region constituted the highest proportion of all three groups. In addition, approximately 98% of participants in all three groups stated that they had a social media account. Regarding the social media platforms to which participants were subscribed, Instagram ranked first in all three groups, followed by Facebook. Finally, approximately 62% of participants stated that they used social media tools in the recruitment process.

**Table 2:** Demographics of participants and distribution across groups.

Variables		Groups					
		Control group *n* = 177		Experiment 1 group *n* = 147		Experiment 2 group *n* = 156	
		*n*	%	*n*	%	*n*	%
Sex	Female	95	53.7	79	53.7	87	55.8
	Male	82	46.3	68	46.3	69	44.2
Age	18–27	15	8.5	13	8.8	13	8.3
	28–37	91	51.4	78	53.1	92	59.0
	38–47	57	32.2	41	27.9	38	24.4
	48 and above	14	7.9	15	10.2	13	8.3
Education level	High school	38	21.5	21	14.3	29	18.6
	Associate	34	19.2	28	19	41	26.3
	Undergraduate	93	52.5	91	61.9	77	49.4
	Graduate	12	6.8	7	4.8	9	5.8
Tenure	Less than a year	27	15.3	16	10.9	23	14.7
	1–4 years	61	34.5	51	34.7	42	26.9
	5–8 years	49	27.7	42	28.6	46	29.5
	7–9 years	20	11.3	21	14.3	33	21.2
	13 years or more	20	11.3	17	11.6	12	7.7
Position	General Manager	27	15.3	22	15	30	19.2
	Deputy General Manager	20	11.3	16	10.9	18	11.5
	HR Manager	68	38.4	59	40.1	60	38.5
	HR Specialist	62	35	50	34	48	30.8
Region	Mediterranean	107	60.8	81	55.1	100	64.1
	Central Anatolia	10	5.7	8	5.4	7	4.5
	Marmara	20	11.4	23	15.6	25	16
	Aegean	25	14.2	25	17	18	11.5
	Black Sea	4	2.3	4	2.7	2	1.3
	Eastern Anatolia	4	2.3	2	1.4	3	1.9
	Southeast Anatolia	6	3.4	4	2.7	1	0.6
Social media account	Yes	175	98.9	144	98.0	156	100
	No	7	4.1	5	3.4	5	3.2
Social media memberships*	Facebook	136	76.7	112	76.2	127	81.4
	Instagram	180	93.1	137	85	149	95.4
	LinkedIn	89	51.2	72	61.7	81	51.9
	Twitter	50	29.1	50	35.5	43	27.5
	Tik Tok	24	14	26	17.8	15	9.6
	None	2	1.2	1	0.7	0	0
Use of social media in the recruitment process	Yes	113	63.8	90	61.2	99	63.5
	No	64	36.2	57	38.8	57	36.5

* Multiple responses were marked by participants.

### 4.2 Outer model

Before proceeding with the analysis of the structural model of the study, validity and reliability analyses of the measurement model were conducted. The analysis results for the measurement model are presented in Table 3. Accordingly, all items in the measurement model are expected to have factor loadings of 0.70 or higher (Hair et al., 2013). Examination of the table results shows that the factor loadings of all items ranged between 0.748 and 0.943. In addition, the T-values for the items were found to be high and statistically significant, ranging from 19.885 to 143.181 (*p* < 0.001). These results indicate that the items loaded strongly under their respective factors and that convergent validity was achieved. When examining the average variance extracted (AVE) values, which represent another criterion for convergent validity, the values obtained for all factors were found to be above the commonly accepted threshold of 0.50 (Hair et al., 2019). This indicates that the factors explain a substantial portion of the variance of their respective scale items, thereby meeting the convergent validity criterion. On the other hand, the Cronbach’s alpha coefficients and composite reliability (CR) values of the scales were found to be above the recommended threshold of 0.70 for all factors. The high values support that the scales achieved internal consistency and reliability.

**Table 3:** Research model.

Items	Path coefficients	T statistics	*p*-values	Cronbach alpha	CR	AVE
Candidate A professional competence 1	0.823	47.632	0.000	0.850	0.905	0.616
Candidate A professional competence 2	0.833	51.565	0.000			
Candidate A professional competence 3	0.755	23.675	0.000			
Candidate A professional competence 4	0.748	20.801	0.000			
Candidate A professional competence 5	0.763	29.828	0.000			
Candidate A person-organization fit 1	0.908	92.401	0.000	0.938	0.938	0.842
Candidate A person-organization fit 2	0.905	88.643	0.000			
Candidate A person-organization fit 3	0.926	114.879	0.000			
Candidate A person-organization fit 4	0.932	143.181	0.000			
Candidate A hiring intention 1	0.924	119.171	0.000	0.895	0.897	0.826
Candidate A hiring intention 2	0.908	97.633	0.000			
Candidate A hiring intention 3	0.895	70.747	0.000			
Candidate B professional competence 1	0.770	22.620	0.000	0.857	0.866	0.636
Candidate B professional competence 2	0.773	19.885	0.000			
Candidate B professional competence 3	0.776	21.692	0.000			
Candidate B professional competence 4	0.810	25.664	0.000			
Candidate B professional competence 5	0.855	45.023	0.000			
Candidate B person-organization fit 1	0.913	89.391	0.000	0.941	0.943	0.851
Candidate B person-organization fit 2	0.902	89.755	0.000			
Candidate B person-organization fit 3	0.931	121.730	0.000			
Candidate B person-organization fit 4	0.943	135.086	0.000			
Candidate B recruitment intention 1	0.908	80.931	0.000	0.890	0.892	0.820
Candidate B recruitment intention 2	0.896	79.142	0.000			
Candidate B recruitment intention 3	0.912	88.003	0.000			

In the study, discriminant validity of the measurement model was evaluated using the Fornell–Larcker and Heterotrait–Monotrait (HTMT) criteria. The results are presented in Table 3. First, according to the Fornell–Larcker criterion, the square root of each factor’s AVE value is expected to be higher than its cross-correlation values (Fornell and Larcker, 1981), and the results in Table 4 show that this condition was met. Therefore, discriminant validity was achieved according to the Fornell–Larcker criterion. Second, the Heterotrait–Monotrait ratio (HTMT) values were examined. HTMT values are generally expected to remain below 0.90 (Henseler et al., 2015). Examination of the HTMT values in Table 3 shows that all values were below the recommended threshold of 0.90. The results indicate that the scales used in the study achieved discriminant validity.

**Table 4:** Results of discriminant validity.

Fornell-Larcker criterion	1	2	3	4	5	6
1. Hiring intention_(a)_	0.909					
2. Professional competence_(a)_	0.377	0.785				
3. Person-organization fit_(a)_	0.772	0.421	0.918			
4. Hiring intention _(b)_	−0.274	−0.208	−0.265	0.906		
5. Professional competence _(b)_	0.073	0.148	0.007	0.294	0.798	
6. Person-organization fit _(b)_	−0.262	−0.163	−0.315	0.774	0.190	0.922

HTMT	1	2	3	4	5	6
1. Hiring intention _(a)_						
2. Professional competence _(a)_	0.400					
3. Person-organization fit _(a)_	0.841	0.433				
4. Hiring intention _(b)_	0.309	0.220	0.290			
5. Professional competence _(b)_	0.089	0.184	0.055	0.333		
6. Person-organization fit _(b)_	0.285	0.157	0.336	0.842	0.209	

(a): Candidate A, (b): Candidate B.

### 4.3 Testing hypothesis

#### 4.3.1 The effect of professional competence and person-organization fit on recruitment intentions

The findings in Table 5 reveal the effect of candidates’ professional competence on evaluators’ hiring intentions. Accordingly, Candidate A’s Professional Competence does not have a significant effect on Hiring Intention (*β* = 0.050, *p* = 0.143). Candidate B’s Professional Competence positively affects Hiring Intention (*β* = 0.175, *p* = 0.000). A strong and significant relationship was found between Candidate A’s Person–Organization Fit and Hiring Intention (*β* = 0.740, *p* = 0.000). This result indicates that the perception of person–organization fit is extremely critical in hiring decisions for a candidate with standard qualifications. Similarly, the strong and significant relationship between Candidate B’s Person–Organization Fit and Hiring Intention (*β* = 0.725, *p* = 0.000) shows that person–organization fit also decisively influences hiring intention for a highly qualified candidate. Based on these results, Hypothesis H1 was partially supported, and Hypothesis H2 was strongly supported.

**Table 5:** Path analysis results for hiring intention.

	Path coefficients	T statistics	*P-*values	*R*^2^	*F*^2^	*Q*^2^
PC_(a)_ → HI_(a)_	0.050	1.464	0.143	0.600	0.005	0.596
POF _(a)_ → HI _(a)_	0.740	24.562	0.000		1.047	
POF_(b)_ → HI _(a)_	−0.033	1.057	0.290		0.002	
PC_(b)_ → HI _(a)_	−0.067	2.071	0.038		0.010	
PC_(b)_ → HI_(b)_	0.175	4.945	0.000	0.631	0.078	0.627
POF _(b)_ → HI_(b)_	0.725	23.995	0.000		1.235	
PC_(a)_ → HI _(b)_	0.122	4.148	0.000		0.032	
KÖU_(a)_ → İAN_(b)_	0.013	0.467	0.641		0.000	

(a): Candidate A, (b): Candidate B, HI: Hiring Intention, POF: Person-Organization Fit, PC: Professional Competence.

#### 4.3.2 Evaluation of candidates by groups

The evaluation results of the control and experimental group evaluators regarding the candidates’ professional competence, person–organization fit, and hiring intentions were compared using one-way ANOVA analysis as can be seen in Table 6. The findings show that there are significant differences between the control and experimental groups in terms of candidates’ professional competence, person–organization fit, and hiring intentions.

**Table 6:** Evaluation results of candidates in control and experimental groups by group (one-way ANOVA analysis results).

Variables\Groups	**Candidate A**						**Candidate B**					
	Control (*n* = 177)	Experimental 1 (*n* = 147)	Experimental 2 (*n* = 156)	*F*	*P*	*Post-hoc*	Control (*n* = 177)	Experimental 1 (*n* = 147)	Experimental 2 (*n* = 156)	*F*	*P*	*Post hoc*
	Mean	Mean	Mean				Mean	Mean	Mean			
Professional competence (a)	3.66	3.49	3.90	14.363	*p* < 0.001	a^c^,b^c^,c^ab^	4.49	4.67	4.34	13.196	p < 0.001	a^b,c^,b^a,c^,c^a,b^
Person-organization fit (b)	3.86	2.92	4.18	100.160	*p* < 0.001	a^b,c^,b^a,c^,c^a,b^	3.88	4.47	3.35	67.516	p < 0.001	a^b,c^,b^a,c^,c^a,b^
Hiring intention (c)	3.80	3.09	4.33	81.896	*p* < 0.001	a^b,c^,b^a,c^,c^a,b^	4.48	4.69	3.60	92.277	p < 0.001	a^bc^,b^ac,^c^a,b^

^a^Professional competence.

^b^Person–organization fit.

^c^Hiring intention.

##### 4.3.2.1 Candidate A (standard qualifications) evaluation results

In terms of Professional Competence, the mean score in the control group was 3.66, while in Experimental Group 1, where negative social media content was presented, this score dropped to 3.49 and in Experimental Group 2, where positive social media content was presented, it increased to 3.90 (*F* = 14.36, *p* < 0.001). This finding indicates that an unprofessional social media profile negatively affects perceptions of a candidate’s technical competence, whereas a professional profile has a positive effect. For the Person–Organization Fit variable, the differences between the experimental groups were much more pronounced (*F* = 100.16, *p* < 0.001). While the average fit score in the control group was 3.86, this value dropped to 2.92 in Experimental Group 1 (negative social media profile) and reached the highest level at 4.18 in Experimental Group 2 (professional social media profile). The results reveal that social media content strongly alters the perception of organizational fit. Hiring Intention followed a similar pattern (*F* = 81.90, *p* < 0.001). The intention score, which was 3.80 in the control group, dropped to 3.09 in Experimental Group 1, while it rose to 4.33 in Experimental Group 2. This finding shows that a candidate with a positive social media profile has a significantly higher likelihood of being hired, whereas a negative profile substantially decreases this likelihood.

##### 4.3.2.2 Candidate B (high qualifications) evaluation results

In the Professional Competence assessment, the mean score in the control group was 4.49, rising to 4.67 in Experimental Group 1, where a positive social media profile was presented, and dropping to 4.34 in Experimental Group 2, where a negative profile was presented (*F* = 13.20, *p* < 0.001). This indicates that a social media profile can influence perceptions of technical competence even for highly qualified candidates. When examining Person–Organization Fit scores, the value in the control group was 3.88, increasing to 4.47 in Experimental Group 1 and decreasing to 3.35 in Experimental Group 2 (*F* = 67.52, *p* < 0.001). A positive social media profile strengthened perceptions of person–organization fit, whereas a negative profile weakened them. For Hiring Intention, the scores were 4.48 in the control group, 4.69 in Experimental Group 1, and 3.60 in Experimental Group 2 (*F* = 92.28, *p* < 0.001). This finding shows that social media content can significantly alter the likelihood of hiring even highly qualified candidates, and that negative content creates a considerable disadvantage. *Post hoc* analyses indicated that both Experimental Group 1 and Experimental Group 2 differed significantly from the control group and also from each other.

The paired-samples t-test results presented in Table 7 allow for a systematic comparison of the evaluation scores of Candidate A (standard qualifications) and Candidate B (high qualifications) within the same group. The analyses show that, in both the control and experimental conditions, there are significant differences between the candidates in terms of the evaluation criteria.

**Table 7:** Paired-sample *t*-test results for the comparison of candidates in control and experimental groups.

Groups	Control Group (n = 177)						Experimental Group 1 (n = 147)						Experimental Group 2 (n = 156)					
	Candidate A		Candidate B				Candidate A		Candidate B				Candidate A		Candidate B			
Variables	Ort.	S.S	Ort.	S.S	*t*	*p*	Ort.	S.S	Ort.	S.S	*t*	*p*	Ort.	S.S	Ort.	S.S	*t*	*p*
Professional competence	3.66	0.744	4.49	0.577	−12.312	*p* < 0.001	3.49	0.755	4.67	0.476	−17.965	*p* < 0.001	3.90	0.514	4.34	0.625	−9.052	*p* < 0.001
Person-organization fit	3.86	0.069	3.88	0.798	−0.199	0.842	2.92	1.061	4.47	0.548	−15.835	*p* < 0.001	4.18	0.630	3.35	1.064	7.352	*p* < 0.001
Hiring intention	3.80	0.732	4.48	0.664	−10.621	*p* < 0.001	3.09	1.124	4.69	0.433	−16.301	*p* < 0.001	4.33	0.634	3.60	1.023	6.561	*p* < 0.001

##### 4.3.2.3 Control group results

In the control group, candidates were evaluated solely based on their résumés. According to the results in Table 7, in terms of Professional Competence, Candidate B, with high qualifications (*x̄* = 4.49), was rated significantly higher than Candidate A, with standard qualifications (*x̄* = 3.66) (*t* = −12.312; *p* < 0.001). This indicates that participants clearly distinguished between the candidates when assessing them based only on their résumés. For Person–Organization Fit, there was no statistically significant difference between Candidate A (*x̄* = 3.86) and Candidate B (*x̄* = 3.88) (*t* = −0.199; *p* = 0.842). This finding suggests that résumé information alone does not create a notable difference in terms of perceived person–organization fit. Regarding Hiring Intention, Candidate B, with high qualifications (*x̄* = 4.48), was significantly more preferred than Candidate A, with standard qualifications (*x̄* = 3.80) (*t* = −10.621; *p* < 0.001). This result shows that higher professional qualifications presented in the résumé have a clear impact on the hiring decision.

##### 4.3.2.4 Experimental group 1 results

In terms of Professional Competence, Candidate B, who possessed high qualifications and a professional social media profile (*x̄* = 4.67), scored significantly higher than Candidate A, who had standard qualifications and a negative social media profile (*x̄* = 3.49) (*t* = −17.965; *p* < 0.001). This finding indicates that social media content amplifies perceptions of professional competence. For Person–Organization Fit, Candidate B with a professional social media profile (*x̄* = 4.47) received much higher scores than Candidate A with an unprofessional social media profile (*x̄* = 2.92) (*t* = −15.835; *p* < 0.001). This result shows that candidates’ social media content strongly shapes evaluators’ perceptions of person–organization fit. Similarly, in terms of Hiring Intention, Candidate B with professional social media content (*x̄* = 4.69) had a clear advantage over Candidate A with a negative profile (*x̄* = 3.09) (*t* = −16.301; *p* < 0.001).

##### 4.3.2.5 Experimental group 2 results

In terms of Professional Competence, Candidate B, who had high qualifications but a negative social media profile (*x̄* = 4.34), still scored higher than Candidate A, who had standard qualifications and a professional profile (*x̄* = 3.90) (*t* = −9.052; *p* < 0.001). However, this gap was smaller than in other group comparisons, indicating that while social media content can negatively influence the evaluation of highly qualified candidates, perceptions of professional competence remain relatively resistant to such effects. For Person–Organization Fit, Candidate A with a professional social media profile and standard qualifications (*x̄* = 4.18) was rated significantly higher than Candidate B with high qualifications but an unprofessional profile (*x̄* = 3.35) (*t* = 7.352; *p* < 0.001). This finding demonstrates that a social media profile can be strong enough to reverse perceptions of person–organization fit. A similar trend was observed for Hiring Intention. Candidate A with a professional social media profile (*x̄* = 4.33) scored significantly higher than Candidate B with a negative profile (*x̄* = 3.60) (*t* = 6.561; *p* < 0.001).

#### 4.3.3 The moderating effect of social media content on evaluators’ hiring intentions

The analysis results in Table 8 show that social media content significantly transforms the candidate evaluation process. For Candidate A, who had standard qualifications, professional competence did not significantly predict hiring intention in either the Control Group or in Experimental Groups 1 and 2 (*p* > 0.05). In contrast, person–organization fit consistently emerged as a strong and reliable predictor across all three conditions (*β*_Control = 0.436; β_Exp1 = 0.775; β_Exp2 = 0.624; *p* < 0.001). Notably, in Experimental Group 1, where a negative social media profile was presented, the coefficient for person–organization fit increased by nearly 80 percent, suggesting that evaluators placed greater emphasis on signals related to cultural fit within the organization.

**Table 8:** Result of moderating effect of social media content.

	Group	Path coefficients	*T* statistics	*P-*values	Bias corrected		Welch-Satterthwait Results
					LB	UB	
PC_(a)_ →HI_(a)_	Control	−0.079	0.807	0.419	−0.229	0.135	C vs. E1: *p* = 0.258
	Experimental 1	0.054	0.834	0.404	−0.073	0.182	E1 vs. E2: *p* = 0.616
	Experimental 2	0.102	1.433	0.152	−0.036	0.239	C vs. E2: *p* = 0.136
PC_(b)_ → HI_(b)_	Control	0.786	23.536	0.000	0.718	0.849	C vs. E1: p = 0.000
	Experimental 1	0.277	2.987	0.003	0.104	0.469	E1 vs. E2: *p* = 0.063
	Experimental 2	0.077	1.438	0.150	−0.036	0.174	C vs. E2: p = 0.000
POF_(a)_ → HI_(a)_	Control	0.436	4.435	0.000	0.204	0.588	C vs. E1: *p* = 0.002
	Experimental 1	0.775	15.240	0.000	0.668	0.867	E1 vs. E2: *p* = 0.106
	Experimental 2	0.624	8.034	0.000	0.460	0.759	C vs. E2: *p* = 0.133
POF_(b)_ → HI_(b)_	Control	0.119	2.811	0.005	0.031	0.196	C vs. E1: *p* = 0.000
	Experimental 1	0.455	5.461	0.000	0.284	0.614	E1 vs. E2: p = 0.000
	Experimental 2	0.865	31.058	0.000	0.805	0.914	C vs. E2: p = 0.000
PC_(a)_ → HI_(b)_	Control	0.016	0.295	0.768	−0.115	0.099	C vs. E1: *p* = 0.522
	Experimental 1	−0.043	0.574	0.566	−0.189	0.109	E1 vs. E2: *p* = 0.111
	Experimental 2	−0.189	3.633	0.000	−0.289	−0.087	C vs. E2: *p* = 0.006
POF_(a)_ → HI_(b)_	Control	−0.014	0.347	0.729	−0.087	0.073	C vs. E1: *p* = 0.084
	Experimental 1	0.113	1.858	0.063	−0.003	0.232	E1 vs. E2: *p* = 0.471
	Experimental 2	0.059	1.345	0.179	−0.029	0.148	C vs. E2: *p* = 0.223
PC_(b)_ → HI_(a)_	Control	0.179	1.759	0.079	0.007	0.405	C vs. E1: *p* = 0.233
	Experimental 1	0.041	0.730	0.465	−0.071	0.151	E1 vs. E2: *p* = 0.945
	Experimental 2	0.034	0.491	0.623	−0.095	0.180	C vs. E2: *p* = 0.242
POF_(b)_ → HI_(a)_	Control	0.134	1.538	0.124	−0.056	0.283	C vs. E1: *p* = 0.334
	Experimental 1	0.035	0.631	0.528	−0.071	0.141	E1 vs. E2: *p* = 0.048
	Experimental 2	−0.133	2.071	0.038	0.012	0.364	C vs. E2: *p* = 0.014

(a): Candidate A, (b): Candidate B, C: Control Group, E1: Experimental Group 1, E2: Experimental Group 2, HI: Hiring Intention, POF: Person-Organization Fit, PC: Professional Competence.

For Candidate B, who had high qualifications, the pattern noticeably shifted. In the Control Group, hiring intention was shaped almost entirely by professional competence (*β* = 0.786, *p* < 0.001). However, when a positive social media profile was presented (Exp1), the effect of professional competence was reduced by almost half (*β* = 0.277, *p* < 0.05), while person–organization fit moderately increased in influence (*β* = 0.455). Even more clearly, with a negative social media profile (Exp2), the coefficient for professional competence lost significance (*β* = 0.077, *p* = 0.150), and person–organization fit became the dominant predictor (*β* = 0.865, *p* < 0.001).

The analysis results in Table 8 indicate that social media’s role in weakening the “competence signal” and strengthening the “fit signal” is also evident in cross-candidate effects. In Experimental Condition 2, Candidate A, who had standard qualifications but a professional social media profile, significantly reduced the acceptance likelihood of Candidate B, who had high qualifications but negative social media content, through a higher professional competence score (*β* = −0.189, *p* < 0.001). In the reverse direction, the same negative social media content triggered a lower perception of person–organization fit for Candidate B, which in turn increased the likelihood of Candidate A being preferred (*β* = −0.133, *p* = 0.038). Welch–Satterthwaite comparisons revealed that the professional competence coefficient for the high-qualified candidate was significantly lower in both experimental groups compared with the control group (*p* < 0.001), while person–organization fit coefficients increased systematically (*p* ≤ 0.002).

## 5 Discussion and implications

This study was designed around the question: *“How do candidates’ social media contents influence evaluators’ hiring decisions?”* The aim was to determine the extent to which and the ways in which the content displayed on candidates’ social media profiles affects decision-makers’ evaluations during recruitment processes. To this end, an experimental study was conducted with managers and human resources specialists who are competent in recruitment within the tourism sector. The experimental method was chosen because it allows the identification of the causal effects of social media content on hiring decisions. In the study, a fictional scenario was developed to create a realistic recruitment situation. Participants were presented with candidates whose résumés differed and whose social media content was manipulated to appear either professional or unprofessional. In this way, the study tested the relative weight of professional qualifications and experience stated in the résumés alongside the personal signals perceived from social media posts in the evaluators’ decision-making processes.

### 5.1 The effect of professional competence and person-organization fit

The first finding of this study indicates that the influence of candidates’ perceived professional competence and person–organization fit on hiring intention varies according to the candidates’ qualification level and the social media content presented (control and experimental groups). For candidates with standard qualifications, professional competence did not have a significant effect on hiring intention. In contrast, for highly qualified candidates, professional competence had a strong and positive effect on evaluators’ hiring decisions. This result shows that professional competence plays a decisive role for highly qualified candidates.

Existing studies also emphasize that professional competence of candidates is an important factor in hiring decisions (Stanišić and Čerović, 2020; Marneros et al., 2021; Derco and Tometzová, 2023). On the other hand, the findings of this study reveal a different circumstance for candidates who have standard professional competence levels. Especially within the framework of signaling theory (Spence, 1973), a candidate’s professional competence (e.g., education, experience, technical skills) constitutes one of the most visible and reliable signals for employers regarding the candidate’s ability (Connelly et al., 2011). However, the extent to which this signal determines hiring decisions is linked to the overall qualification level of the candidate. For highly qualified candidates, competence signals (such as advanced education, prestigious work experience, multiple foreign languages, or advanced technical skills) provide reassurance that the candidate will exceed job requirements, reducing employer uncertainty and increasing the likelihood of selection.

By contrast, for candidates with standard qualifications, professional competence usually meets only the minimum expectations and can be easily substituted by other applicants. In such cases, competence does not create sufficient differentiation to influence the decision significantly. In other words, having standard-level competence is a necessary condition for suitability but not, by itself, a sufficient reason for selection (Līce and Sloka, 2019). Research in the hospitality sector also suggests that professional competence is not limited to technical skills but also involves managerial abilities, cultural sensitivity, and communication skills (Kwok et al., 2011; Marneros et al., 2021; Derco and Tometzová, 2023). While high competence in a highly qualified candidate functions as a strong and decisive signal, in a standard candidate it remains weaker and less decisive. This goes to show that professional competence in hiring decisions is evaluated contextually and in conjunction with the candidate’s overall qualification level, and what creates significant difference is its “excellence” over its “existence.”

Another finding of the study is that the perception of person–organization fit has a strong and significant effect on hiring decisions for both the standard-qualified candidate (Candidate A) and the highly qualified candidate (Candidate B). In particular, while professional competence was not a determining factor in the hiring decisions for the standard-qualified candidate, person–organization fit significantly and strongly influenced such decisions. Similarly, for the highly qualified candidate, person–organization fit emerged as a decisive factor in hiring decisions. These results suggest that in recruitment processes, the perceived person–organization fit of a candidate can have a stronger impact than professional competence.

Person–organization fit is defined as the degree of alignment between an individual’s values, attitudes, and behaviors and the organization’s values, norms, and culture (Kristof, 1996). For employers, this alignment means not only selecting a suitable employee but also gaining someone who will contribute to the organizational culture, demonstrate strong commitment, and have a low likelihood of turnover (Chatman, 1989; Cable and Judge, 1996). The findings of this study indicate that employers place considerable emphasis not only on technical competencies but also on the candidate’s potential to integrate into the organization.

Existing studies consistently show that employees with a high degree of person–organization fit experience greater job satisfaction, exhibit stronger organizational commitment, and have lower turnover intentions (O'Reilly et al., 1991; Kristof-Brown et al., 2005). This reduces recruitment costs while enhancing employee productivity and performance. In sectors such as tourism, where human interaction is intensively observed, the ability of employees not only to perform their tasks technically but also to work harmoniously within teams, build effective relationships with customers, and represent the organizational culture is of critical importance (Marneros et al., 2021; Derco and Tometzová, 2023; Barakazı, 2023). While professional competence is often seen as a skill that can be learned and developed, personality, values, and cultural fit are viewed as more enduring and difficult to change. For this reason, employers may consider that technical gaps can be addressed through training, whereas cultural misalignment could harm both the individual and the organization. The initial findings of this study therefore suggest that organizations prefer candidates who not only meet job requirements but also contribute culturally and can sustain a long-term, mutually beneficial relationship with the workplace.

### 5.2 The effect of social media content

The findings show that the content displayed in candidates’ social media profiles plays a strong moderating role in shaping the influence of professional competence and person–organization fit on hiring decisions. For the standard-qualified Candidate A, professional competence did not significantly predict hiring intention under any condition, while person–organization fit consistently emerged as a strong determinant across all three scenarios. Notably, in Experiment 1, where Candidate A was presented with a negative social media profile, the coefficient for person–organization fit increased significantly. This suggests that evaluators tend to prioritize cultural fit over technical competence when making use of signals from social media content, thereby shaping their hiring intentions accordingly.

In contrast, the results for the highly qualified Candidate B differ markedly. In the control group, hiring inclination was largely driven by professional competence. However, the most striking finding appeared in the condition with negative social media content. Here, the coefficient for professional competence lost statistical significance, while person–organization fit became the most dominant predictor. This indicates that social media content can weaken the impact of technical competence signals even for highly qualified candidates, leading evaluators to place greater emphasis on cultural fit signals when forming their hiring decisions.

The results obtained show that professional competence influences the intention to hire only for the highly qualified candidate when the social media content is positive; when the content turns negative, person–organization fit becomes the primary criterion for both candidates. The social media profile functions as a strong moderator that either reinforces (professional content) or weakens (unprofessional content) the impact of the technical signals presented in the résumé. In this way, evaluations of candidates’ professional competence and person–organization fit are significantly framed through social media, and this framing plays a decisive role in the hiring intention. Therefore, social media profiles emerge not only as a complementary source of information but also as a strategic signaling mechanism that guides the decision-making process and deeply influences the perceived suitability of candidates.

In the Turkish context, these findings suggest that cultural fit is a dominant criterion in hiring decisions. Türkiye’s collectivist cultural structure (Hofstede, 2001; Fikret-Pasa et al., 2001) leads employers to place considerable emphasis not only on candidates’ technical skills but also on the extent to which they align with the organization’s values, norms, and social fabric. Indeed, studies conducted within the Turkish business context demonstrate that managers often regard alignment with organizational values as at least as important as, and in some cases more important than, technical competence when making selection decisions (Aycan, 2001; Barakazı, 2023; Bilgin and Kutlu, 2022).

According to Signaling Theory in the recruitment context, employers do not rely solely on the information provided in résumés and technical documents; they also seek alternative sources of signals to gain insight into candidates’ unobservable attributes. Social media profiles play an important role at this point (Bangerter et al., 2012; Roulin and Bangerter, 2013; Bohmova and Pavlicek, 2015; McDonald et al., 2022; Demir and Günaydın, 2023). Social media makes candidates’ values, behaviors, social relationships, and cultural fit potential visible, enabling employers to gain a more comprehensive and often more personal impression of the candidate. Professional social media content produces strong signals that the candidate’s values, attitudes, and social behaviors align with the organizational culture, while unprofessional content calls this alignment into question and leads to negative evaluations (Kwok et al., 2011; Barakazı, 2023). In this context, a professional and carefully curated social media profile functions as a positive signal, indicating not only that the candidate possesses the technical competencies required for the job but also that they can adapt to the organization’s social and cultural structure.

By contrast, unprofessional and inappropriate social media content can overshadow a candidate’s professional competence signals and create negative impressions among evaluators. In the context of positive–negative information asymmetry (Baumeister et al., 2001), people tend to pay more attention to negative information than to positive information and perceive it as more powerful. This mechanism is also valid in the hiring context. Negative content shared by a candidate on social media (e.g., inappropriate language, excessive partying, behaviors that contradict corporate values) can exert a strong negative influence in evaluations and outweigh the candidate’s technical qualifications. Studies in the literature point to similar results. Research by Becton et al. (2019), Kanar et al. (2010), and Özkum (2018) shows that unprofessional content on social media profiles reduces evaluation scores regardless of the candidate’s competencies, whereas professional content generates only a small positive effect. This explains why negative signals on social media are perceived as stronger than positive ones and influence hiring intentions more markedly. From the perspective of Signaling Theory, social media profiles provide signals not only about candidates’ technical qualifications but also about less observable attributes—such as person–organization fit and personality traits (Connelly et al., 2011). Thus, professional content sends positive fit signals, whereas negative content generates low-status and low-fit signals that can weaken hiring intentions.

However, the findings may also suggest that certain cognitive biases play a role in evaluators’ decision-making mechanisms. In particular, when the “negativity bias” (Baumeister et al., 2001) is at play, negative social media content may overshadow professional competence signals to some extent, and evaluators may tend to shift their decision weights in this direction. Evaluators may be more sensitive to negative cues encountered on social media and interpret these cues as strong—though not always definitive—signals that relegate professional competence to a secondary role. Furthermore, in the Turkish organizational context, where the concepts of “representing the organization” and “reputation” are highly valued (Fikret-Pasa et al., 2001), negative signals derived from social media may become relatively more salient in evaluative processes. Nonetheless, it should also be acknowledged that these effects may vary across different sectors, job positions, and organizational cultures.

In conclusion, candidates’ social media content appears to play a strong moderating role capable of altering evaluators’ decisions in recruitment processes. While professional content can highlight candidates’ strengths, unprofessional content can generate negative perceptions even for highly qualified candidates. In particular, the strong asymmetric impact of negative content and the way information gathered from social media triggers evaluators’ rapid decision-making mechanisms show that social media is an influential tool in candidate assessment. The findings of this study indicate that employers and human resource professionals evaluate social media content not merely as supplementary information but as a significant source of signals that shape hiring decisions.

### 5.3 Theoretical contributions

The findings of this study make contributions to the literature, particularly within the framework of Signaling Theory (Spence, 1973). In the labor market, employers face incomplete information about candidates’ suitability and qualifications and therefore seek to reduce uncertainty by using signals such as résumés, education, and work experience. Our findings show that, in addition to the information in résumés, social media profiles generate new signals that reflect not only what candidates post but also their potential alignment with organizational culture. In this sense, social media emerges as a new and powerful source of signals, conveying not only candidates’ technical competencies but also their values, attitudes, and potential cultural fit. The results are also consistent with the view that signals transmitted via social media can have a strong impact in both positive and negative directions (Becton et al., 2019; Alarcon et al., 2019; McDonald et al., 2022). Negative social media content was found to reduce hiring intentions even when candidates possessed high professional competence, and this effect proved stronger than the positive effect generated by professional content. This finding aligns with the “negativity bias” literature in social psychology (Baumeister et al., 2001). The results further demonstrate that negative social media content can almost eliminate the effect of professional competence on hiring intentions, while significantly increasing the weight of person–organization fit. Thus, the study provides empirical evidence that signals transmitted via social media can moderate the influence of signals contained in résumés. This finding contributes to Signaling Theory by clarifying which type of signal is prioritized under specific contextual conditions.

### 5.4 Practical implications

#### 5.4.1 For job seekers

In addition to its theoretical contributions, this study offers important practical implications regarding the use of social media in recruitment processes. Alongside traditional résumés, it is evident that employers and human resource professionals actively use social media during hiring. First, in today’s context, it is critical for candidates to have a professional social media account to showcase their skills, experience, and personal brand. Existing studies show that employers tend to evaluate candidates without a social media profile negatively (Alexander et al., 2019; Johnson, 2023). A social media profile constructed with consistent and accurate information helps employers verify the accuracy of the information provided by the candidate and creates a positive impression. Social media profiles are considered part of candidates’ online identities, and highlighting professionalism on these platforms can offer an advantage during recruitment.

Studies reveal that many social media users are unaware of the potential consequences of their posts (Cain, 2011). Employers view certain types of content positively, such as professional posts aligned with the position, hobbies demonstrating versatility, sports activities, participation in charitable events and social responsibility projects, and awards received. In contrast, inappropriate or provocative photographs, excessive alcohol or drug use, negative remarks about previous employers, colleagues, or clients, and discriminatory statements are perceived negatively and can adversely affect hiring decisions.

A social media profile constructed with consistent and accurate information assists employers in verifying the reliability of the candidate’s claims and provides a favorable first impression. Social media profiles are regarded as an extension of candidates’ online identities; therefore, reflecting professionalism, work ethic, and alignment with organizational values on these platforms can create a distinct advantage in the recruitment process. At this point, candidates are advised to generate professional content (e.g., work-related achievements, professional development activities, participation in social responsibility initiatives), avoid content that may create negative perceptions, and ensure that the experiences and qualifications listed in their résumés are accurately and consistently reflected on their social media profiles. Roulin and Bangerter (2013) and Berkelaar and Buzzanell (2014) note that the information contained in candidates’ social media profiles often provides “more honest signals” of their abilities and commitment. This is because candidates are typically less aware that their profiles may be scrutinized by employers, and therefore, social media content is subject to less impression management than résumés. In line with these findings, it can be argued that job seekers should view their social media profiles as an extension of their résumés (Baert, 2018) and evaluate every piece of publicly shared content through a professional lens.

#### 5.4.2 For organizations and managers

The inclusion of social media data in recruitment decisions can provide organizations with up-to-date, rich, and multidimensional information about candidates. However, the use of such data also entails various legal, ethical, and methodological risks. Therefore, it is of critical importance to develop clear and standardized human resource policies that ensure a fair, consistent, and transparent approach to the integration of social media data into recruitment processes (Jeske and Shultz, 2016; McFarland and Ployhart, 2015). Such policies should explicitly define the types of information that may be considered in hiring decisions (e.g., hate speech, disclosure of confidential information, professional achievements), specify how candidates’ social media profiles will be reviewed, and determine the extent to which the information obtained will be incorporated into the decision-making process. Moreover, these policies must comply with personal data protection regulations (such as KVKK and GDPR) and anti-discrimination principles. The purpose of data processing, the duration of data retention, and access rights must be clearly delineated in order to safeguard candidates’ privacy rights while also protecting organizations from potential legal risks. In this respect, the development of such policies not only ensures standardization in practice but also serves as a legal safeguard for organizations.

While social media has the potential to reveal personal aspects of candidates that are not reflected in their résumés, such information may be misinterpreted or evaluated in a biased manner, leading to unfair outcomes. For example, posts made by candidates in the past may no longer reflect their current attitudes or values. Furthermore, cognitive biases—such as the weighting of negative information (“negativity bias”) or the halo/horn effect—can undermine the objectivity of evaluations. Therefore, it is important that human resource professionals receive regular training on bias awareness, cognitive distortions, and anti-discrimination practices. Such training will contribute to more conscious, fair, and ethical use of social media data in recruitment processes. In conclusion, the use of social media data in recruitment involves both significant opportunities and substantial risks. The accurate interpretation of candidates’ digital identities, the implementation of policies grounded in ethical principles, the enhancement of HR professionals’ bias awareness, and the adoption of transparent practices are essential to this process. This approach will support organizations in making effective recruitment decisions while also fostering a fair and ethical evaluation environment for candidates.

## 6 Recommendations for future studies

Although this study confirmed through an experimental design that social media serves as a signaling mechanism in hiring decisions, new lines of research are needed to enhance the generalizability of the findings and deepen theoretical explanatory power. Our research focused on the tourism sector, specifically on the sales and marketing manager position. Similar experimental protocols could be applied in highly regulated sectors such as healthcare, finance, and information technology, as well as across different job levels including blue-collar positions, to test the sectoral sensitivity of social media signals. In the tourism field, comparing positions with varying degrees of customer interaction such as front office, food and beverage, or entertainment would reveal how the priority order between professional competence and cultural signals changes according to job type. In the present experiment, only male candidate profiles were used. It is recommended to include profiles of female candidates and, if possible, those from different ethnic or demographic groups to examine the effects of gender and bias. This would allow an assessment of whether social media signals are perceived with different weight depending on gender. Studies conducted with multi-country samples on perceptions of social media content could test whether certain signals (e.g., alcohol-related photographs, political posts) are universal or culturally specific, thereby questioning the cultural validity of the theoretical framework. The proposed directions would present the role of social media signals in recruitment processes from a more comprehensive and cross-cultural perspective, offering both theoretical enrichment and practical guidance for practitioners.

## Data Availability

The raw data supporting the conclusions of this article will be made available by the authors, without undue reservation.
